# Design of the **P**acemaker **RE**mote **F**ollow-up **E**valuation and **R**eview (PREFER) trial to assess the clinical value of the remote pacemaker interrogation in the management of pacemaker patients

**DOI:** 10.1186/1745-6215-9-18

**Published:** 2008-04-03

**Authors:** Jane Chen, Bruce L Wilkoff, Wassim Choucair, Todd J Cohen, George H Crossley, W Ben Johnson, Luc R Mongeon, Gerald A Serwer, Lou Sherfesee

**Affiliations:** 1 Department of Internal Medicine, Cardiovascular Division, Washington University School of Medicine, Saint Louis, Missouri, USA; 2 Department of Cardiovascular Medicine, Cleveland Clinic, Cleveland, Ohio, USA; 3 Cardiology Associates of Corpus Christi, Corpus Christi, Texas, USA; 4 Department of Medicine, Winthrop University Hospital, Mineola, New York, USA; 5 Electrophysiology Department, St. Thomas Research Institute, Nashville, Tennessee, USA; 6 Iowa Heart Center, Des Moines, Iowa, USA; 7 Medtronic, Inc, Minneapolis, Minnesota, USA; 8 Department of Pediatrics, University of Michigan Congenital Heart Center, University of Michigan Health System, Ann Arbor, Michigan, USA

## Abstract

**Background:**

Although pacemakers are primarily used for the treatment of bradycardia, diagnostic data available in current pacemakers allow them to be also used as sophisticated, continuous monitoring devices. Easy access to these stored data may assist clinicians in making diagnostic and therapeutic decisions sooner, thus avoiding potential long-term sequelae due to untreated clinical disorders. Internet-based remote device interrogation systems provide clinicians with frequent and complete access to stored data in pacemakers. In addition to monitoring device function, remote monitors may be a helpful tool in assisting physicians in the management of common arrhythmia disorders.

**Methods:**

The **P**acemaker **RE**mote **F**ollow-up **E**valuation and **R**eview (PREFER) trial is a prospective, randomized, parallel, unblinded, multicenter, open label clinical trial to determine the utility of remote pacemaker interrogation in the earlier diagnosis of clinically actionable events compared to the existing practice of transtelephonic monitoring. There have been 980 patients enrolled and randomized to receive pacemaker follow up with either remote interrogation using the Medtronic CareLink^® ^Network (CareLink) versus the conventional method of transtelephonic monitoring (TTM) in addition to periodic in-person interrogation and programming evaluations. The purpose of this manuscript is to describe the design of the PREFER trial. The results, to be presented separately, will characterize the number of clinically actionable events as a result of pacemaker follow-up using remote interrogation instead of TTM.

**Trial registration:**

ClinicalTrials.gov: NCT00294645.

## Background

Pacemaker therapy is indicated to treat bradycardia due to sinus node or atrioventricular conduction disorders. In addition to pacing, current devices have expanded recording and diagnostic capabilities, providing continuous cardiac monitoring and long-term trended clinical information. Pacemakers can store atrial as well as ventricular high rate episodes, along with percentage of atrial and ventricular pacing, capture management data, and many other clinically useful parameters. Furthermore, the atrial high rate diagnostic data in pacemakers have been shown to have good sensitivity and specificity for the diagnosis of atrial fibrillation (AF) [1,2]. Pacemaker therapy monitoring is most frequently achieved in the United States with a combination of in-person programming evaluations and transtelephonic monitoring (TTM). TTMs are primarily used to monitor battery status, and are performed at a frequency adjusted as implant duration progresses. TTMs, however, have no ability to download the monitored data stored in pacemakers. Full device data interrogations are only available through in-person programming evaluations.

The advent of internet-based remote monitoring systems allow for full device interrogation while the patient is in their home or other remote location. This allows clinicians to have more frequent and complete access to the device data without creating a substantial burden on the patient and the clinician with increased office visits. In addition to the Medtronic CareLink^® ^Network (CareLink) (Minneapolis, MN, USA), remote monitoring systems are also available through Biotronik Home Monitoring (Berlin, Germany), St. Jude Housecall™ (St. Paul, MN, USA), and Boston Scientific Latitude^® ^(St. Paul, MN, USA). The functionality of these systems has been well described for use in patients with implantable cardioverter-defibrillators (ICD). Clinically significant findings such as ventricular tachycardia [3], silent AF [4], early volume overload [5], and lead fractures causing inappropriate shocks [6] have been detected during clinicians' reviews of the remote transmissions. Remote monitoring systems, however, have not been described for use in patients with pacemakers.

The purpose of the PREFER trial is to evaluate the utility of systematic remote interrogation to monitor pacemaker and patient condition in order to increase the awareness of physicians to any events which may necessitate early clinical intervention.

## Methods/Design

### Hypothesis

The PREFER trial examines the hypothesis that quarterly device monitoring by remote interrogation will result in the earlier diagnosis of clinically actionable events compared to the combination of TTM and routine office visits.

### Primary endpoint

The primary endpoint is the rate of first diagnosis of clinically actionable events in patients whose pacemakers are followed by remote interrogation versus those whose pacemakers are followed by office visits augmented by TTM. The goal of early identification of clinically actionable events is to have early intervention by a clinician, which could impact the clinical sequelae. The clinically actionable events were selected on the basis that their presence merits a clinical decision or further medical assessment. Clinically actionable events are defined as:

• Atrial tachycardia (AT)/AF episodes ≥ 48 hours (defined as two consecutive days in which the device records at least 18 hours of AT/AF per day)

• Ventricular pacing that has increased by 30 percent relative since last device interrogation

• Sensed ventricular rate of ≥ 100 bpm during atrial arrhythmia for at least 20 percent of the time since previous device interrogation

• Non-sustained ventricular tachycardia (NSVT) ≥ 5 beats

• New onset AT/AF in patients with no prior history of AT/AF

• Loss of capture

• Increase in pacing voltage threshold ≥ 1 V

• Significant changes in atrial or ventricular lead impedance, defined as:

◦ < 200 of > 2000 ohms

◦ Unstable lead impedance deemed to be clinically actionable

◦ ≥ 50 percent change in lead impedance since last interrogation

• Elective Replacement Indicator (ERI) or End of Life (EOL)

### Secondary endpoints

The first secondary endpoint of the trial is to characterize the frequency of actions for each of the clinically actionable events defined above. There are nine additional secondary objectives; each assesses the contribution of individual clinically actionable events to the primary objective.

### Design

The PREFER trial is a prospective, randomized, parallel, unblinded, multicenter, open label clinical trial to investigate the clinical value of remote interrogation in the management of patients with pacemakers. All study patients received a Medtronic Kappa^® ^900, EnPulse^®^, Adapta™, or Versa™ device supported by CareLink. The pacemakers are either single- or dual-chamber devices. Fifty investigative centers in the United States (US) enrolled a total of 980 patients between May 2004 and March 2007, with planned 12-month follow up.

The primary inclusion criteria are:

◦ Patient is at least 30 days post system modification, including new device implant, device upgrade, or lead changes.

◦ Patient has access to an analog phone line.

◦ Patient is capable of operating the TTM monitor and the CareLink Monitor.

The exclusion criteria are:

◦ Enrollment in another pacemaker clinical study that might confound the results of this trial.

◦ Patient is a candidate for an ICD.

All patients who met eligibility requirements and signed an Institutional Review Board (IRB) approved PREFER trial Informed Consent Form and an Authorization for the Use and Disclosure of Health Information underwent an in-office baseline evaluation to quantify baseline characteristics. Baseline evaluations included pacemaker information, cardiovascular medical and surgical history, New York Heart Association (NYHA) classification, and arrhythmia history.

### Randomization

**Figure 1:**
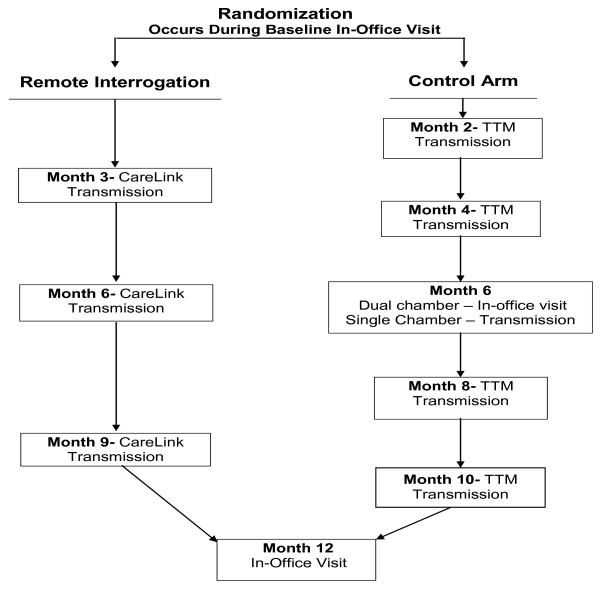
**Trial design flowchart**. Trial design.

Patients were randomized 2:1 to remote interrogation at three month intervals or TTM evaluations at two month intervals. A permutated block randomization was used to randomize the subjects. Patients who were randomized to follow up with the remote interrogation will transmit pacemaker information at 3, 6, and 9 months after enrollment and have an office visit at 12 months post-enrollment. The TTM control subjects with single chamber devices will transmit 30 second TTM strips with and without magnet application at 2, 4, 6, 8, and 10 months after enrollment and have an office visit at 12 months. Patients with dual chamber devices will transmit via TTM at 2, 4, 8, and 10 months after enrollment, with office visits at 6 and 12 months post enrollment. The frequency of TTM transmissions is the maximal permitted under Centers for Medicare and Medicaid Services (CMS) reimbursement guidelines [7] and reflective of average frequency of TTM transmissions from Medicare patients. The trial design flowchart is shown in Figure 1.

### Programming requirements

The objective of the programming requirements for the PREFER trial is to detect AF (as inferred by atrial high rate episodes), and/or ventricular high rates for either AF with rapid ventricular response rate or ventricular tachycardia. Specific requirements are listed in Table 1.

**Table 1:** Programming requirements

**Parameter**	**Value**
Atrial high rate episode	Rolling
Ventricular high rate episode	Rolling
Ventricular minimum detection duration rate	5 beats

### Data collection

All scheduled and unscheduled transmissions will be recorded. Designated physicians and nurses at the clinical site will determine if a clinically actionable event can be diagnosed based on the transmitted data. Additional information will be collected at each office visit, including medication changes and the performance of device interrogations. The data collected and stored during interrogations consists of an evaluation of thresholds, lead impedance, battery voltage, as well as a review of device diagnostics, and stored electrograms.

### Statistical methods and data analysis

#### Sample size methods and assumptions

Data from the Atrial High Rate (A-HIRATE) study were used to estimate the survival rates for patients in each arm of the PREFER trial. The A-HIRATE study evaluated pacemaker patients implanted with a Kappa 700 or Kappa 900 device [8]. Due to device diagnostic limitations of the Kappa 700, only the 157 subjects in the A-HIRATE study that were implanted with a Kappa 900 device and followed through at least one month post-implant were used for event rate estimation, as this most closely approximates patients in the PREFER trial.

Simulations were used to determine the sample size necessary to evaluate the primary objective while following patients through quarterly remote interrogations compared to the combination of TTM transmissions and in-office visits, while achieving 80 percent power and α = 0.05. A sample size requirement of 700 subjects was determined.

#### Analysis of primary objective

The time to first diagnosis of a clinically actionable event or censoring will be determined for each randomized subject. The Peto & Peto modification of the Gehan-Wilcoxon test will be performed [9]. Only events diagnosed by the clinician will count toward the primary endpoint. If the test yields a p-value less than 0.05, it will be concluded that the freedom from first diagnosis of clinically actionable event(s) is significantly lower when patients are followed through remote interrogation as they were in this trial, compared to being followed through TTM and scheduled in-office visits.

#### Analysis of secondary objectives

The same analysis will be repeated for each of the individual events that make up the composite primary endpoint. In addition, actions taken in response to the clinician awareness of these events will be summarized.

#### Supplemental analyses

Subsequent to verification of database accuracy and closure, an examination of the distribution of each study endpoint will be performed. Descriptive statistics for each demographic variable will be calculated which include measures of average, variability, frequency, and a count of the number of missing values. No data imputation will be performed. When the mean is found not to be an appropriate measure of central tendency, alternative statistics will be considered (e.g. median).

The distribution of demographic and clinical background parameters will be summarized for the study population overall and for each relevant study group.

Subjects in the remote interrogation arm of the trial will be asked to complete a patient survey at the 12 month visit in order to characterize the burden of in-office follow-up assumed by patients and their family. The survey questions include mileage traveled and time spent by the patient to reach the office, type of transportation used, necessity of the patient to take time off of work for the office appointment, and time spent by any family members in order to bring patient to the office. Descriptive statistics will be computed to summarize the results of the questionnaire.

## Discussion

The PREFER trial hypothesized that the systematic remote interrogation allows clinicians earlier and more complete access to pacemaker diagnostic data than TTM and scheduled in-office visits. If the results support this hypothesis, events such as ventricular response rate in AF, presence of NSVT, rising lead thresholds, and significant changes in lead integrity and battery status, will therefore be detected earlier so that earlier clinical actions and decisions may then take place. Thus, the pacemaker monitoring system can become much more a part of clinical decision and management.

One of the most common arrhythmias encountered in pacemaker patients is AF. In a study by Gillis et al of incidence of AF in pacemaker patients, 15 percent of patients who developed AF as documented on their pacemakers did not have AF pre-implant [10]. In the A-HIRATE trial, 46 percent of patients without a history of AF developed at least one atrial high rate episode (AHRE) by 24 months [8]. Because symptoms are often inaccurate [11] and patients are frequently asymptomatic in AF, earlier identification of AF by pacemakers, with frequent access to the data via remote monitors, can mean earlier initiation of appropriate anticoagulation which may reduce stroke events.

The relationship of AF burden to stroke risks remains unclear. In an ancillary study from the MOde Selection Trial (MOST), patients with atrial high rate episodes (AHRE) greater than 5 minutes had twice the death and stroke rates as patients without any AHRE [2]. In another study, patients with AHRE greater than 1 day had a 3-fold increase in strokes compared with patients with AHRE less than a day [12]. The continuous monitoring capabilities of pacemakers may assist in determining exactly the association between the amount of AF burden and stroke rates. The TRENDS study, which aimed to study the relationship between duration of AHRE and stroke events in patients with AF and who are not anticoagulated, has completed enrollment and is currently in analysis phase, and will be providing more data [13]. The ASSERT study is also underway and will evaluate whether the detection of AHRE with pacemaker telemetry predicts an increased risk of stroke and other vascular events [14]. If a relationship does exist between the duration of AF and stroke risks, then the ability to diagnose AF as soon as possible via a remote monitoring system could indeed be a clinically valuable tool.

Other advantages of remote monitoring of pacemakers include the ability to access rate histograms. In patients with known AF, rate control is crucial to avoid tachycardia-mediated cardiomyopathy, and frequent remote access to rate histograms can allow clinicians to better monitor the success of rate control. Additionally, the potential detrimental effects of right ventricular pacing have gained prominence in recent years. A substudy from MOST demonstrated an increased risk of heart failure hospitalization in patients who had over 40 percent ventricular pacing [15]. The incidence of AF also increased with right ventricular (RV) pacing. Pacemakers now document the percentage of RV pacing delivered, thus allowing clinicians to make programming changes to limit the amount of RV pacing. Ventricular high rate episodes recorded by pacemaker may also suggest NSVT, which may be an indication of cardiomyopathy in patients not previously suspected to have structural heart disease. Appropriate screening can then be performed to assess the patients' risks for sudden cardiac death and candidacy for upgrade to an ICD.

Remote monitoring systems are in widespread use for monitoring ICDs, but have not yet been studied for the monitoring of pacemakers. To our knowledge, this is the first large, multi-centered trial to compare the utility of remote monitoring systems with the existing system of TTM and office visits. It is our hypothesis that pacemaker patients can develop clinically significant arrhythmic issues, that pacemaker diagnostic data are sophisticated and numerous, and that frequent access to these data via remote monitoring can result in earlier actions by clinicians.

## Competing interests

Dr. Chen, Dr. Wilkoff, Dr. Cohen, Dr. Crossley, Dr. Johnson, and Dr. Serwer have received reimbursements, fees, funding or salary from Medtronic. Mongeon and Sherfesee are salaried Medtronic employees. Dr. Choucair has declared no competing interests.

## Authors' contributions

JC made significant contributions the drafting, revising the manuscript, and provided final approval of version to be published. BLW is Principle Investigator for the study described in the manuscript and made significant contributions to study design, drafting, revising the manuscript, and provided final approval of version to be published. WC participated in revising the manuscript and review prior to final approval of version to be published. TJC participated in revising the manuscript and review prior to final approval of version to be published. GC participated in revising the manuscript and review prior to final approval of version to be published. BJ participated in revising the manuscript and review prior to final approval of version to be published. LM participated in development of the study concept, revisions to the manuscript and review of the final version to be published. GS participated in revising the manuscript and review prior to final approval of version to be published. LS performed statistical analysis, participated in modification to the study design, drafting and revising the manuscript, and participated in review of the final version to be published.
